# Assessment of Platelet Mitochondrial Respiration in a Pediatric Population: A Pilot Study in Healthy Children and Children with Acute Lymphoblastic Leukemia

**DOI:** 10.3390/children8121196

**Published:** 2021-12-17

**Authors:** Theia Lelcu, Anca M. Bînă, Maria D. Dănilă, Călin M. Popoiu, Oana M. Aburel, Smaranda T. Arghirescu, Claudia Borza, Danina M. Muntean

**Affiliations:** 1 Department III Functional Sciences, Discipline Pathophysiology, “Victor Babeș” University of Medicine and Pharmacy, E. Murgu Sq. No. 2, 300041 Timișoara, Romania; lelcu.theia@umft.ro (T.L.); lungu.anca@umft.ro (A.M.B.); danila.maria@umft.ro (M.D.D.); oanaduicu@umft.ro (O.M.A.); daninamuntean@umft.ro (D.M.M.); 2 Centre for Translational Research and Systems Medicine, “Victor Babeș” University of Medicine and Pharmacy, E. Murgu Sq. No. 2, 300041 Timișoara, Romania; 3 Department XI Pediatrics, Discipline Pediatric Surgery, “Victor Babeș” University of Medicine and Pharmacy, E. Murgu Sq. No. 2, 300041 Timișoara, Romania; mcpopoiu@umft.ro; 4 Department XI Pediatrics, Discipline Pediatrics III, “Victor Babeș” University of Medicine and Pharmacy, E. Murgu Sq. No. 2, 300041 Timișoara, Romania; 5 “Louis Țurcanu” Emergency Hospital for Children, 300011 Timișoara, Romania

**Keywords:** platelets, high-resolution respirometry, children, age-dependency of mitochondrial respiration, acute lymphoblastic leukemia

## Abstract

Characterization of mitochondrial respiration in peripheral blood cells has recently emerged as a potential biomarker for the assessment of the severity of hematological malignancies (HM) in adults. Whether changes in platelet respiratory function occur in children with or without HM it is unknown. The present pilot study was double-aimed: (i) to investigate whether platelet respiration is age-dependent in non-HM children and (ii) to assess the platelet mitochondrial respiration in children with newly diagnosed acute lymphoblastic leukemia (ALL). Blood samples obtained from age-grouped children (10–11, 13–14 and 16–17 years) with non-HM and children with ALL (10–11 years) were used to isolate platelets via differential centrifugation. High-resolution respirometry studies of isolated platelets were performed according to a protocol adapted to evaluate complex I and II-supported respiration. An age-related decrease in respiration was observed in the non-HM pediatric population and had comparable values for the 13–14 and 16–17 years. groups. In children with ALL, a significant increase in C I-supported active respiration and decrease in maximal noncoupled respiration were found at the disease onset. In conclusion, in a pediatric population, platelet mitochondrial respiration is age-dependent. Platelet respiratory dysfunction occurs in children with newly-diagnosed ALL, an observation that warrants further investigation of this change as a disease biomarker.

## 1. Introduction

Mitochondrial dysfunction has emerged in the past few decades as the central pathomechanism in a plethora of acute and chronic diseases [1,2,3]. Assessment of the cellular energetic profile by means of novel research techniques allows a better understanding of the disease pathogenesis, progression and therapeutic response [4,5].

Platelets are metabolically active fragments derived from megakariocytes with versatile roles in the complex interplay of key pathophysiological processes, such as chronic inflammation, hemostasis and thrombosis, and immune modulation in cancer [6].

Recent studies have revealed that assessment of mitochondrial function in peripheral blood cells may become a valid biomarker of disease and also a potential indicator of the overall metabolic health [1,2,3,4,5]. Accordingly, mitochondrial respiratory dysfunction in platelets and mononuclear cells has been reported to occur in various chronic and acute pathologies [1,7,8,9,10]. High-resolution respirometry is a sensitive method for assessing mitochondrial respiration in tiny amounts of human samples including blood cells [4,11,12,13,14]. Platelets are important sources of viable mitochondria in humans and can be sampled in a less invasive manner as compared to other organs, such as skeletal muscle, which require biopsy [2,4,9]. A recent elegant study by Baaten et al. described the mitochondrial dysfunction in platelets isolated from patients diagnosed with various hematological malignancies (HM) and chemotherapy-induced thrombocytopenia [15]. Whether changes in platelet mitochondrial respiration occur in pediatric patients with newly-diagnosed HM has not been investigated so far. Moreover, literature data regarding the age-dependency of platelet mitochondrial respiration are scarce [2].

The present study performed in a pilot pediatric group was aimed: (i) firstly, to investigate the influence of age on platelet respiration in children with non-hematological pathology, and (ii) second, to determine the platelet respiratory parameters in children with newly-diagnosed acute lymphoblastic leukemia (ALL), the most frequent HM in pediatrics.

## 2. Materials and Methods

### 2.1. Study Population

Children were recruited from the “Louis Țurcanu” Emergency Hospital for Children, Timișoara, Romania, the Clinic of Pediatric Surgery (for the non-HM group) and the 3rd Clinic of Pediatrics (for the HM group), respectively. The study was conducted in accordance with the statements of the Declaration of Helsinki and the protocol was approved by the Committee of Research Ethics of “*Victor Babeș*” University of Medicine and Pharmacy, Timișoara, Romania (nr. 42/20.12.2018). Written informed consent was provided by all the parents or tutors of the included children after the experimental procedure was explained.

### 2.2. Sample Acquisition and Platelet Preparation

Peripheral blood was sampled from the control group, the non-HM children (*n* = 6, two for each age-groups, 10–11 years, 13–14 years and 16–17 years, respectively) and children with newly-diagnosed ALL (10–11 years, *n* = 4), i.e., prior to any therapeutic intervention (i.e., chemotherapy, antibiotic therapy, transfusion therapy or radiotherapy) after their parents or tutors signed the informed consent. The exclusion criteria of the ALL group were: altered clinical state, severe anemia, severe thrombocytopenia, sepsis, organ dysfunction or disseminated intravascular coagulation). The non-HM children were hospitalized for minor elective surgical interventions (varicocele and hernia) or post-fracture rehabilitation and the exclusion criteria were: altered clinical state, emergency interventions (e.g., appendicitis associated with acute inflammation), severe anemia or sepsis. Peripheral blood (10 mL) was sampled via a single venous puncture in K_2_ EDTA tubes (BD Vacutainer) and immediately transferred to the laboratory. Platelet isolation was performed within maximum 4 h after sampling. Freshly isolated platelets were immediately used for high-resolution respirometry (HRR) studies.

Platelet isolation was performed according to a previously described protocol [16]. Blood was centrifuged at room temperature at 500× *g* in order to obtain platelet rich plasma that was further centrifuged at 4600× *g*. The resulting platelet pellet was resuspended in own plasma and the number of platelets was counted with a blood analyzer (Sysmex^®^) and used to calculate the number to be added in the chambers of the O2-k-oxygraphs in order to have 50 million cells/milliliter. The number of isolated platelets/microliter of plasma was 820.333 +/− 63.398 in the non-HM group vs. 328.750 +/− 63.783 in the HM one since the platelet pellet was reduced in the diseased vs. control group.

### 2.3. Characteristics of the Patients with ALL

The patients included in the HM group (*n* = 4) were children (10–11 years) diagnosed with ALL. At the admission they presented with pallor, enlarged lymph nodes and hepato-splenomegaly and a satisfactory overall clinical state. They were afebrile and no signs of infections were detected. After peripheral blood sampling for HRR studies, bone marrow and cerebrospinal fluid (CSF) analysis was performed. Bone marrow aspiration was performed, and the diagnosis was established according to the flow-cytometry. Central nervous system leukemia was excluded for all the patients after CSF analysis (Pandy reaction, microscopy and cell count). Genetic testing was subsequently performed in order to include these patients in the corresponding risk group. Treatment and risk stratification was initiated according to the chemotherapy treatment protocol ALL-IC-BFM 2009 (International BMF–Berlin-Frankfurt-Münster Study Group) [17]. Individual patient’s characteristics are described in Table 1. Based on genomic characteristics, immunophenotype and treatment response a single patient was included in the high-risk group, according to the treatment protocol [17].

### 2.4. High-Resolution Respirometry Studies

Mitochondrial respiration was measured at 37 °C using the Oxygraph-2k (Oroboros Instruments GmbH Innsbruck, AT, Austria) in the mitochondrial respiration medium MIRO5 (0.5 mm EGTA, 3 mm MgCl_2_, 60 mm K-lactobionate, 20 mm taurine, 10 mm KH_2_PO_4_, 20 mm HEPES, 110 mm sucrose and 1 g/L bovine serum albumin, pH 7.1) [11].

For HRR measurements, platelets (50 million cells/milliliter) were suspended in MIRO5 and permeabilized with digitonin (1 μg/1 × 10^6^ platelets), with the subsequent evaluation of the electron transport system (ETS) function, as previously described [2]. Mitochondrial respiration was assessed according to a classical Substrate-Uncoupler-Inhibitor-Titration (SUIT) protocol [16]. Platelet oxygen consumption was allowed to stabilize until a steady state (ROUTINE respiration) was reached. Subsequently, the following additions were made: (1) glutamate (G, 5 mm) and malate (M, 5 mm)-the complex I (C I) substrates, and ADP (1 mm) to measure the active respiration or OXPHOS capacity driven by C I (OXPHOS_C I_); (2) succinate (S, 10 mm)-the complex II (C II) substrate, to induce the maximal active respiration (OXPHOS CAPACITY, P); (3) oligomycin, the ATP synthase inhibitor (1 μg/mL) to assess the non-phosphorylating respiration (a dissipative component of mitochondrial respiration known as LEAK); (4) FCCP (carbonyl cyanide p-trifluoro-methoxy phenyl-hydrazone), a classical uncoupler, was further titrated in successive steps (1 μM/titration step) to measure the maximal non-coupled respiration or the convergent respiratory capacity of the electron transport system (ETS CAPACITY, E); (5) rotenone, the C I inhibitor (2 μM), to assess the ETS capacity supported only by C II (ETS_C II_); (6) in the end, mitochondrial respiration was inhibited by the addition of complex III inhibitor antimycin A (1 μg/mL), allowing the measurement of residual oxygen consumption (ROX). All values of the respiratory parameters were corrected for ROX and used for further analysis [18].

The following flux-control ratios related to coupling were calculated according to refs. [16,19]:

- P-L control efficiency—evaluates the efficiency of ATP generation and was calculated by subtracting LEAK respiration from OXPHOS capacity and then dividing the result by the OXPHOS capacity (P-L/P).
- E-L coupling efficiency—measures the degree of coupling and was calculated by subtracting LEAK respiration from the ETS capacity and then dividing the result by the ETS capacity (E-L/E).
- R-L net routine capacity—is the respiratory capacity available for phosphorylation of ADP to ATP and was calculated as the difference between Routine respiration and LEAK respiration.
- E-L net ET capacity—is the respiratory capacity potentially available for ion transport and phosphorylation of ADP to ATP and was calculated as the difference between ETS capacity and LEAK respiration.
- Routine control ratio—evaluates how close routine respiration (R) operates to the ETS maximal capacity and was calculated as the ratio between Routine and ETS capacity (R/E).

### 2.5. Data Analysis

Statistical analysis was performed with GraphPad PRISM (GraphPad Software version 9.0, La Jolla, CA, USA). Data are presented as means ± SEM. A one-way ANOVA with post hoc analysis by Tukey’s multiple comparison test was used to compare multiple groups and *t* test was used to assess differences between non-HM and ALL children. The results were considered statistically significant when the *p* value was below 0.05.

### 2.6. Reagents

All the chemicals used in this study were purchased from Sigma-Aldrich (Merck).

## 3. Results

### 3.1. Mitochondrial Respiration Decreased with Age in Non-HM Children

**Figure 1:**
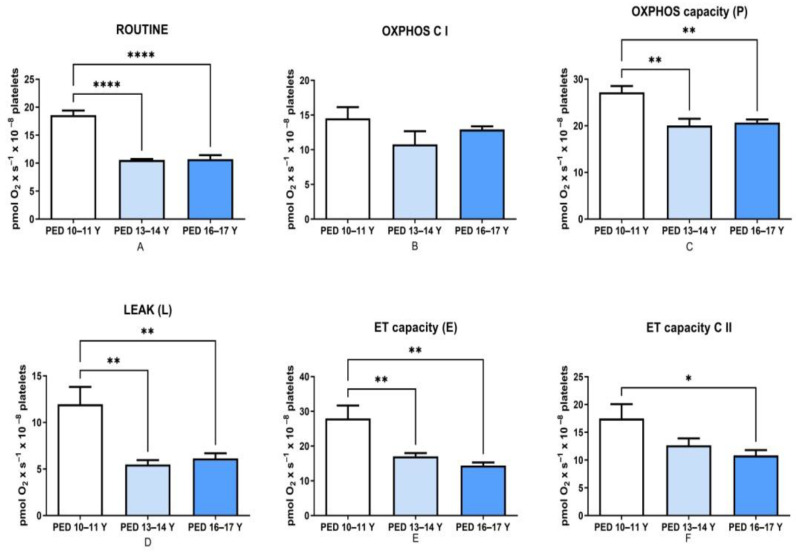
The influence of age on platelet mitochondrial respiratory rates in children with no HM. Data are presented as mean ± SEM (* *p* < 0.05, ** *p* < 0.01, **** *p* < 0.0001). HM: Hematological Malignancies. (**A**). Age-dependency of Routine respiration; (**B**). Age-dependency of complex I-supported active respiration; (**C**). Age-dependency of complex I and II-supported active respiration; (**D**). Age-dependency of non-phosphorylating respiration; (**E**). Age-dependency of the maximal non-coupled respiration supported by complex I and II; (**F**). Age-dependency of the maximal non-coupled respiration supported by complex II. OXPHOS C I: Oxidative Phosphorylation driven by complex I; Maximal OXPHOS (C I and C II): Oxidative Phosphorylation driven by complex I and II; LEAK: non-phosphorylating respiration; ET (Electron Transport) capacity: maximal non-coupled complex I and II-supported respiration; ET capacity C II: maximal non-coupled respiration supported by complex II.

In this pilot study, we report an age-dependent decrease in platelet mitochondrial respiratory rates in children hospitalized for non-HM (Figure 1). The most significant difference was found in the older groups (13–14 Y and 16–17 Y) as compared to the younger one (10–11 Y) for routine respiration (Figure 1A, *p* < 0.0001). As depicted in Figure 1C, the maximal oxidative capacity dependent on both complex I and II (but not the one dependent only on C I–Figure 1B) was significantly decreased in both older groups vs. the 10–11-year-old group, thus suggesting that the change was mainly related to C II-supported respiration. Furthermore, a significant decrease in the non-phosphorylating respiration (LEAK) for both respiratory complexes was found in the above-mentioned older groups vs. the younger one (Figure 1D, *p* < 0.01). The maximal non-coupled C I + C II-dependent respiration (ET CAPACITY, E) was also significantly decreased with the age (Figure 1E, *p* < 0.01). As regarding the non-coupled state depending on complex II, the ET capacity C II decrease was significant only for the oldest group 16–17 as compared to the youngest investigated, suggesting that changes in C II-dependent respiratory rates are more important as children are growing up (Figure 1F, *p* < 0.05).

**Figure 2:**
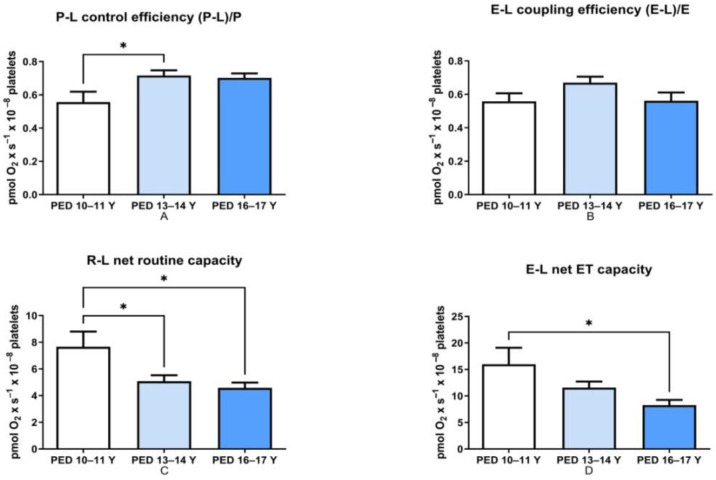
The influence of age on flux-control ratios in the control groups. Data are presented as mean ± SEM (* *p* < 0.05). (**A**). The influence of age on the ATP generation efficiency; (**B**). The influence of age on the degree of coupling; (**C**). The influence of age on the respiratory capacity available for phosphorylation of ADP to ATP; (**D**). The influence of age on the respiratory capacity potentially available for ion transport and phosphorylation of ADP to ATP.

With respect to the flux-control ratios, an increase in the P-L control efficiency (ATP generation) occurred in the two older groups vs. the 10–11-year-old one (even if statistical significance was found only for the 13–14-year-old group (Figure 2A, *p* < 0.05). No age dependency was found regarding the ET coupling efficiency (Figure 2B).

Both the R-L net routine capacity (Figure 2C, *p* < 0.05) and the E-L net ET capacity (Figure 2D, *p* < 0.05) were decreased in the 13–14-year-old and 16–17-year-old groups vs. the 10–11 year-old one. These findings emphasize the importance of selecting appropriate age-matched controls when assessing platelet mitochondrial dysfunction in children.

### 3.2. Platelet Respiration Is Modified at the Onset of ALL

The second objective of the study was to assess platelet mitochondrial respiration of 10–11-year-old children with newly-diagnosed ALL (*n* = 4) as compared to their age-matched peers (Table 2).

In this pilot study, at the onset of disease (i.e., prior to any therapeutic intervention), we found a significant lower routine respiration (10.8 ± 0.971 vs. 18.6 ± 0.8, *p* < 0.0001). The marked decrease in routine respiration elicited a significant decrease in the R/E ratio (0.545 ± 0.05 vs. 0.737 ± 0.09, *p* < 0.05).

The decrease in non-phosphorylating (LEAK) respiration was also observed in children with ALL. Moreover, the maximal convergent respiratory capacity of ETS (E) was significantly low at the onset of the disease (20.8 ± 1.42 vs. 28 ± 3.73, *p* < 0.05). This decrease in mitochondrial oxygen consumption in the fully uncoupled state (after FCCP titration) was, most probably, due to the decrease in ET capacity of complex II (12.2 ± 0.793 vs. 17.5 ± 2.59, *p* < 0.05).

More important, a significant increase (19.3 ± 0.96 vs. 14.5 ± 1.61, *p* < 0.05) in the active respiration dependent on complex I (OXPHOS_C I_) was found in the setting of ALL, thus indicating an early change in the respiratory capacity in the presence of the NADH-generating substrates.

An increasing trend (albeit non-significant) in both P-L control efficiency and in ET coupling efficiency was also observed.

Interestingly, in patient No. 1 with high-risk T-ALL, an important increase in routine and LEAK respiration was observed as compared to the medium risk patients with B-ALL patients (data not shown).

## 4. Discussion

The first major finding of the present pilot study is the age-related decrease in the respiratory rates in children with non-HM. When we recruited the children for the control group, we did not expect an age-dependent variation in platelet respiration. However, when we ranged the respirometry data according to the children’s age, we found a significant difference in platelet mitochondrial respiration between the first age-group (10–11 years) and the other two older groups (13–14 years, and 16–17 years, respectively).

Sjovall et al. first assessed platelet mitochondrial respiration in children (1 month–12 years, with a mean age of 4) and adults (19–65 years, with a mean age of 37) and reported a significant correlations with age, namely an increase in routine respiration and decrease in the maximal uncoupled respiration dependent on complex II [2]. Our data are in line with the latter finding, i.e., we found a decrease in the ET capacity for C II in the oldest group (16–17 years) as compared to the youngest (10–11 years) one. At variance from their results, our preliminary data also show a lower C II-supported OXPHOS capacity in adolescents as compared to the young children. The significance of this age-related decrease in C II-dependent respiratory rates is unknown and requires further investigation.

Previous studies have shown a transient increase in insulin resistance during puberty [20,21,22,23]. Moran et al. performed a cross-sectional analysis of 357 healthy children and adolescents and demonstrated that a physiological, transient insulin resistance occurs at the onset of puberty, which correlates to the rapid growth and the development period [24]. Fleischman et al. assessed the mitochondrial function in skeletal muscle in 74 healthy and overweight children aged between 8 and 18 years, by means of ^31^P magnetic resonance spectroscopy [25]. This non-invasive technique evaluates mitochondrial capacity and provides a clinically relevant measure of mitochondrial function. The authors showed that the decrease in skeletal muscle mitochondrial oxidative phosphorylation was strongly correlated with insulin resistance in children [25]. Whether the age-dependent decrease in platelet mitochondrial respiration found in the present study is caused by a transient insulin resistance that physiologically occurs at puberty deserves further investigation.

The second major finding of the present study is the early impairment of platelet mitochondrial respiration at the onset of ALL in children as compared to their age-matched controls. In particular, a decrease in routine respiration, LEAK and the maximal non-coupled respiration was found. These changes may be related to the: (i) modification in mitochondrial morphology or content, (ii) inhibition of substrate transport across the mitochondrial inner membrane, (iii) impairment of the electron transport chain, or a combination thereof.

At variance, complex-I supported active respiration was increased.

An increasing body of evidence suggests that circulating blood cells can be successfully used for the assessment of mitochondria dysfunction as mirror of organ and tissue mitochondria changes in various pathologies [2,3,26].

However, literature regarding mitochondrial dysfunction in hematological malignancies is scarce. Baaten et al. performed a comprehensive analysis of mitochondrial function (including HRR) and reported the impairment of platelet mitochondrial respiration in adults diagnosed with blood malignancies and treated with chemotherapy. These authors reported that the maximal ADP-supported respiration was significantly decreased in platelets isolated from seven adults diagnosed with blood malignancies, which were previously treated with chemotherapy. Platelet reactivity was not significantly changed until these patients became thrombocytopenic. However, an increase in ROS production and a decrease in the mitochondrial membrane potential were also revealed approximately 10 days after therapy, which may be suggestive of mitochondrial dysfunction elicited by chemotherapy at the level of megakaryocytes [15].

A significant increase in the mitochondrial mass associated with a higher oxygen consumption rates has been previously reported in leukemic cells as compared to normal cells [27].

Over the past few decades, it has become clear that leukemic cells present considerable metabolic plasticity and rely upon an increased mitochondrial function [28]. Most of the studies in this respect have tackled the acute myeloid leukemia (AML).

Basak and Banerjee underlined the importance of mitochondrial OXPHOS in the progression of AML [29]. Farge et al. demonstrated that AML cells, in mouse xenografts models, which possessed a high OXPHOS status are resistant to cytarabine, an essential chemotherapeutic drug in the treatment of AML [30]. Ashton et al. demonstrated in a recent comprehensive review the role of OXPHOS as a novel therapeutic agent in cancer therapy [31]. Most attempts to target the ETS have focused on complex I inhibition. Accordingly, Baccelli et al. demonstrated both in vitro and in vivo the antileukemic role of an ubiquinone-dependent complex I inhibitor. Their study on primary AML cells highlighted the increased sensitivity of cells from poor outcome patients, which were more OXPHOS dependent [32]. Panina et al. have recently summarized in an excellent review the ongoing research regarding the complex I inhibitors (such as metformin and IACS-010759) as adjunct therapy in association with chemotherapeutics in the treatment of AML [28].

We have demonstrated here that an increase in C I-supported OXPHOS occurs in platelets isolated from children with ALL at the moment of diagnosis. Whether this mirrors the events occurring at the level of lymphocytes is unknown. Nevertheless, an upregulation in oxidative phosphorylation has been proven to promote treatment resistance in acute lymphoblastic leukemia [33,34]. In a recent study, Xuedong et al. demonstrated that ALL lymphocytes are characterized by an increased mitochondrial biogenesis as compared to normal hematopoetic and mononuclear cells. Furthermore, they confirmed the role of the antibiotic tigecycline, which inhibits mitochondrial respiration and sensitizes ALL cells to standard chemotherapy drugs [35].

Hlozkova et al. revealed in a recent study in human leukemia cell lines that lymphoid leukemias present lower basal respiration and a preference for oxidative metabolism, which correlated with a high sensitivity to L-Asparaginase, a common drug used in the chemotherapy regimens of ALL [36].

Previous studies suggested a vicious pathophysiological circle in which malignant cells promote oxidative stress within the neighboring cells which represent the tumor microenvironment. The increase in oxidative stress within the tumor microenvironment determines aerobic glycolysis and lead to an increased production of energy substrates such as pyruvate, lactate, ketone bodies, which fuel the mitochondrial OXPHOS of the malignant cells and lead to an efficient ATP production [37].

Not only in acute hematologic malignancies, but also in chronic lymphocytic leukemia and B-cell non-Hodgkin lymphoma, cells primarily rely on OXPHOS [37,38,39]. Caro et al. reported metabolic differences in Diffuse Large B-Cell Lymphomas (DLBCL), with an OXPHOS-DLBCL subtype characterized by elevated oxidative phosphorylation, which may become a druggable target in this HM as well [38]. In a recent study, Chowdbury et al. performed mitochondrial bioenergetics profiling in primary chronic lymphocytic leukemia cells (CLL) and reported that CLL cells from patients with adverse prognostic presented a higher maximal respiration [40].

The fact that an increase in mitochondrial respiration was associated with worse outcome is not new. In a pioneering study from 2010, Sjovall et al. investigated mitochondrial respiration in platelets harvested from patients diagnosed with severe sepsis or septic shock. These authors reported an increase in basal respiration and the fact that platelet mitochondrial uncoupling was paralleled by a progressive and significant increase in respiratory capacity as a compensatory response to severe sepsis [9]. Of note, in the patient with high-risk T-ALL, a significant increase in routine and LEAK respiration was observed when compared to MR (medium risk) group of B-ALL.

Several mechanisms have been reported to account for the platelet abnormalities in acute leukemias. Firstly, the uncontrolled proliferation of leukemic blasts is responsible for both the replacement of normal bone marrow cells with leukemic cells and suppression of megakaryocytes production [41].

A parameter used to assess platelet production in the bone marrow is the immature platelet fraction percentage (IPF%). These immature platelets are larger and still contain residual ARN. Strauss et al. have reported that children with newly diagnosed ALL presented a higher IPF%, which means that during leukemogenesis, the thrombopoiesis process is initially accelerated. They assumed that platelet thrombopoiesis in ALL may also take place in other areas such as lymph nodes, reticuloendothelial system, spleen or liver. However, when analyzing the IPF% after the initiation of chemotherapy, patients with ALL who undergo treatment have a decreased IPF% [42].

In the search for noninvasive biomarkers for the early diagnosis of pediatric ALL by assessing serum proteomic profiles, Shi et al. reported that platelet factor–4 (PF4), an indicator of megakaryocyte maturation and differentiation, and platelet activation and pro-platelet basic protein precursor (PBP) were found to be down-regulated in ALL patients [43].

Furthermore, several transcription factors such as RUNX1, GATA1, FLI1, GFI1B, MECOM, ETV6, and NFE2 are implicated in megakaryopoiesis and platelet biogenesis. Both ETV6 and RUNX1 are known regulators of hematopoiesis [44]. In pediatric malignancies, the *ETV6-RUNX1 (TEL-AML1)* fusion gene is the most common chromosomal alteration, which occurs in approximately 25% of children with B cell precursor-acute lymphoblastic leukemia [45]. It is tempting to speculate that a defect in the transcription factors which regulate platelet biogenesis (ETV6, RUNX1), and which occurs in pediatric ALL, may affect platelet mitochondrial respiration.

Velez et al. investigated the effect of platelets on mitochondrial respiration and its impact on leukemogenesis. They used both AML and ALL cell lines and demonstrated that leukemic cells determine platelet activation. Moreover, when exposed to platelet lysate, both types of leukemic cell lines showed an increased mitochondrial uncoupling that reduced the basal and stimulated-superoxide production and determined an increased stability and a higher intrinsic apoptotic resistance, thus promoting leukemogenesis and resistance to treatment [46].

Last but not least, inflammation associated with an altered cytokine production is another pathophysiological event present at the onset of disease in ALL. Horachek et al. assessed the correlation between prognostic factors, overall survival and event-free survival and the serum levels of certain cytokines (soluble receptor α for IL-2, soluble receptor for IL-6, soluble receptor for TNF-α type I and soluble receptor for TNF-α type II) in adults diagnosed with B-cell precursor acute lymphoblastic leukemia [47].

Whether the changes in platelet respiration reported in children with newly-diagnosed ALL are also due to the impaired thrombopoiesis and inflammatory reaction and cytokine response at the beginning of the leukemogenic process has not been assessed in the present study. Moreover, we acknowledge as a limitation of the present study the reduced size of the groups (due to the emerging COVID-19 pandemics); therefore, a larger confirmatory study is clearly warranted.

## 5. Conclusions

An age-dependent decrease in platelet mitochondrial respiration occurs in children, with comparable values of the respiratory parameters between 13 and 17 years. Mitochondrial respiratory dysfunction in peripheral platelets is present at the moment of diagnosis in 10–11-year-old children with acute lymphoblastic leukemia, an observation that warrants further investigation as a potential early biomarker in this hematological malignancy.

## Figures and Tables

**Table 1:** ALL group patient characteristics.

	Immuno- Phenotype	Risk Group	Karyotype/Genomic Characteristics
Patient 1	Mature T-ALL (Egil stage IV)	High risk (HR)	t(1;14)(p32q11)
Patient 2	Common B ALL	Medium risk (MR)	*TEL-AML1 (ETV6-RUNX1)* positive
Patient 3	Common B ALL	Medium risk (MR)	Normal Karyotype; major *BCR-ABL1*(b2a2-b3a2-Fusion)-negative; minor *BCR-ABL1* (e1a2 Fusion)-negative; *KMT2A-AFF1* (*MLL1-AF4*)-negative; *TCF3-PBX1 (E2A-PBX1)*-negative; *ETV6-RUNX1 (TEL-AML1)*-negative
Patient 4	Pre-B ALL	Medium risk (MR)	Normal Karyotype; major *BCR-ABL1* (b2a2-b3a2-Fusion)-negative; minor *BCR-ABL1* (e1a2 Fusion)-negative; *KMT2A-AFF1 (MLL1-AF4)*-negative; *TCF3-PBX1 (E2A-PBX1)*-negative*; ETV6-RUNX1 (TEL-AML1)*-negative

**Table 2:** Mitochondrial respiratory parameters in children with ALL vs. age-matched non-HM children (details in text).

Respiratory Parameters	ALL Group	Age-Matched Non-HM Group
Routine	10.8 ± 0.67 ****	18.6 ± 0.8
OXPHOS C I	19.3 ± 0.95 *	14.5 ± 1.61
Maximal OXPHOS (C I and II)	27.0 ± 1.42	27.2 ± 1.36
LEAK (L)	7.79 ± 1.25	12 ± 1.85
ET capacity (E)	20.8 ± 1.42 *	28 ± 3.73
ET capacity C II	12.2 ± 0.79 *	17.5 ± 2.59
P-L control efficiency (P-L/P)	19.2 ± 1.67	15.2 ± 1.95
ET coupling efficiency (E-L/E)	0.65 ± 0.054	0.558 ± 0.048
R/E	0.545 ± 0.05 *	0.737 ± 0.09

Data are presented as mean ± SEM (**** *p* < 0.0001, * *p* < 0.05). ALL: Acute Lymphoblastic Leukemia; HM: Hematological Malignancies; OXPHOS C I: oxidative phosphorylation driven by complex I; Maximal OXPHOS (C I and C II): oxidative phosphorylation driven by complex I and II; LEAK: non-phosphorylating respiration; ET capacity (E): maximal non-coupled respiration supported by complex I and II; ET capacity C II: maximal non-coupled respiration supported by complex II; P–L (Phosphorylation—LEAK) coupling efficiency (evaluates the efficiency of ATP generation); ET (Electron Transport system) coupling efficiency (measures the degree of coupling); R/E (Routine/ET) control ratio (evaluates how close routine respiration operates to the ET maximal capacity).

## Data Availability

Data are contained within the article.
